# Tip Effect of the Tapping Mode of Atomic Force Microscope in Viscous Fluid Environments

**DOI:** 10.3390/s150818381

**Published:** 2015-07-28

**Authors:** Hua-Ju Shih, Po-Jen Shih

**Affiliations:** Department of Civil and Environmental Engineering, National University of Kaohsiung, No. 700, Kaohsiung University Rd., Nanzih District, 81148 Kaohsiung, Taiwan; E-Mail: alan1438alan@hotmail.com

**Keywords:** atomic force microscope, pressure, vorticity, semi-analytical method

## Abstract

Atomic force microscope with applicable types of operation in a liquid environment is widely used to scan the contours of biological specimens. The contact mode of operation allows a tip to touch a specimen directly but sometimes it damages the specimen; thus, a tapping mode of operation may replace the contact mode. The tapping mode triggers the cantilever of the microscope approximately at resonance frequencies, and so the tip periodically knocks the specimen. It is well known that the cantilever induces extra liquid pressure that leads to drift in the resonance frequency. Studies have noted that the heights of protein surfaces measured via the tapping mode of an atomic force microscope are ~25% smaller than those measured by other methods. This discrepancy may be attributable to the induced superficial hydrodynamic pressure, which is worth investigating. In this paper, we introduce a semi-analytical method to analyze the pressure distribution of various tip geometries. According to our analysis, the maximum hydrodynamic pressure on the specimen caused by a cone-shaped tip is ~0.5 Pa, which can, for example, pre-deform a cell by several nanometers in compression before the tip taps it. Moreover, the pressure calculated on the surface of the specimen is 20 times larger than the pressure without considering the tip effect; these results have not been motioned in other papers. Dominating factors, such as surface heights of protein surface, mechanical stiffness of protein increasing with loading velocity, and radius of tip affecting the local pressure of specimen, are also addressed in this study.

## 1. Introduction

To investigate biological specimens (bio-specimens) in a liquid environment, atomic force microscope (AFM) is currently the best tool for measuring surface contours [1,2]. Two techniques commonly applied to investigate nano-scalar structures are scanning electron microscopy (SEM) and scanning probe microscopy (SPM). However, AFM has greater applicability than SPM for the observation of bio-specimens in a liquid environment. Additionally, SEM can be problematic when observing specimens containing water, since it must operate in a vacuum chamber. The bio-specimens containing water are scattered or smashed when exposed to vacuum. Thus, we pursue an alternative technique for AFM of bio-specimens.

The two basic modes of AFM operation with respect to probing in a liquid environment are the contact mode and the tapping mode. In the contact mode, a long or low-stiffness cantilever is equipped to prevent damaging bio-specimen in the scanning process [3]. The tapping mode is an advanced technique at which the probe tip touches the specimen only at the end of its downward movement. It reduces the contact time and the friction forces compared to contact mode AFM. The system measures shifts in amplitude or frequency as feedback and control in the scanning process. Compared to contact mode AFM, tapping mode AFM is capable of imaging macromolecule surfaces even when the molecules are only weakly attached to a substrate. However, the vibrating probe produces pressure and vorticity at the edges between the tip and the beam, and these affect the pressure on the specimen. Many studies have discussed the dynamics of probes in a liquid environment [1,4], but few have focused on pressure caused by these tips. We believe the liquid pressure and vorticity induced by the vibrating tip may deform the geometric surface of the specimen, especially when the tip and specimen are close. In this paper, we investigate the effects of tips and the pressure on the topography measurement of bio-specimens, such as hexagonally packed intermediate (HPI) layer surfaces and extracellular/cytoplasmic purple membrane surfaces.

A widely adopted method for investigating the hydrodynamics caused by a vibrating probe in liquid is the boundary integral formulation, which is a semi-analytical method with a strongly theoretical background. Tuck [5] proposed an integral method using infinitely long rigid cylinders with arbitrary cross sections to obtain explicit results. The method has subsequently been applied to practical applications such as AFM and microelectromechanical systems. Sader *et al.* [6,7,8] extended Tuck’s study and used it to calculate the hydrodynamic load on an infinitely long rigid beam of zero thickness in a viscous fluid. Scherer *et al.* [9] adopted this and compared these results with the mechanical impedance in experiments. Green *et al.* [10,11] calculated the added liquid-mass by modifying the damping of the liquid and considering the normal and torsional modes of AFM so that the frequency shifts of the cantilever could be predicted. Alcaraz *et al.* [12] used a microrheological model to analyze the hydrodynamic drag force on the AFM probe and proposed the drag force as a function of tip–specimen distance at low Reynolds number. Additionally, the finite element method is helpful for considering the hydrodynamics of AFM probes, such as the hydrodynamic loading of the microcantilever [13], the cellular strain distributions [14], and the stress at the edge of the specimen [15]. Since researchers have focused on achieving higher resolutions, many papers have focused on the bending mode with higher order terms, the torsional mode, the tip jump, and methods for estimating the added mass. However, few studies have considered problems from the perspective of the specimen; for example, the added mass loads on the specimen when the tip is very close to it.

The tapping mode has become a widely used technique for scanning bio-specimens. When the tip taps the specimen, the system suffers interference from liquid in the small gap between the beam and the specimen. At the same time, both the beam and the tip apply pressure to the liquid, and this pressure is transferred to the specimen. Generally, the topographies measured by the tapping mode and the contact mode is very similar in lateral resolution. However, Möller *et al.* [16] found that averaged heights of the topography of outer HPI layer surfaces and extracellular/cytoplasmic purple membrane surfaces measured by the tapping mode were ~25% smaller than those measured by the contact mode. Medalsy and Müller [17] found that the mechanical stiffness of a protein and membrane could increase by 10 times with the loading velocity. Further, it is known that diseases, like cancer, can induce mechanical property changes in cells. The property may potentially serve as a useful biomarker in the early detection of cancer [18]. Vichare *et al.* addressed the influence of cell geometry and cell pre-stress on cell stiffness measurements [19]. The hydrodynamic pressure may have an impact on the above issues and need clarification.

To quantify the pressure on the surface of the specimen, we focus on the interaction between the tip and the specimen. We use the semi-analytical method to obtain the pressure and the vorticity. Here, the pressures and vorticity are considered only before the tip contacts the specimen. We consider four tip shapes: a cone—shaped tip, a bell—shaped tip, a sharp tip, and a sine-shaped tip. The results show that the tip causes deformation before the tip makes contact with the specimen. Furthermore, the maximum magnitude of pressure is around 0.5 Pa, which is sufficiently large to deform a specimen’s height by several nanometers. Moreover, the pressure calculated on the surface of the specimen is 20 times larger than the pressure without considering the tip effect. The model without considering the tip effect is widely studied in many papers currently [6,7,8,9,10,11].

## 2. Mathematical Model

In this paper, we modified the boundary integral method from that studied by Sader *et al.* [6], and we added the contribution of the presence of a tip in the original model. We make the following six assumptions: (i) a two-dimensional model is considered; (ii) the amplitude of oscillation is small compared with the characteristic lengths of the cantilever; (iii) the fluid is incompressible; (iv) the surfaces on the tip, the cantilever, and the specimen are no-slip; (v) the surface of the specimen is considered a plane when compared with the characteristic scales of the tips; and (vi) the thickness of the cantilever is small when compared with its width. Since the amplitude of the tapping mode of AFM is necessarily small, the assumption that the fluid is incompressible is natural and provides a wavelength of vibration exceeding the cantilever’s characteristic length scales. In addition, the nonlinear convective inertial effects in the fluid are negligible, and so only the linearized unsteady Navier-Stokes equation needs to be considered.

A beam oscillating with small amplitude in liquid has a Fourier-transformed unsteady Navier-Stokes equation:

(1) $$-i\omega \rho u=-\nabla P+\eta \nabla^{2}u$$

(2) ∇·u=0

where **u**(*y*,*z*|ω) is the velocity field with two components (*v*,*w*), *P*(*y*,*z*|*ω*) is the pressure, *ρ* is the density of liquid, ω is the angular velocity, and η is the viscosity. The Fourier transform of a function with respect to time *t* is given by $\hat{x}(\omega )=\int_{-\infty }^{\infty }x(t)e^{i\omega t}dt$. We introduce a stream function *ψ*(*y*,*z*|*ω*) so the fluid velocity is defined by *v*(*y*,*z*|*ω*) = ∂*ψ*(*y*,*z*|*ω*)/∂*z* and in the *y* and *z* direction, respectively. Application of Green’s integral theorem results in the following integral representation of the stream function for the unsteady Stokes equations:

(3) $$\begin{array}{ll} \psi (y^{\prime },z^{\prime }|\omega ) & =\int_{C}[\psi (y,z|\omega )G_{n}(y,z|y^{\prime },z^{\prime })-\psi_{n}(y,z|\omega )\Omega (y,z|y^{\prime },z^{\prime })\\ & -\zeta \;\Psi_{n}(y,z|y^{\prime },z\prime )+P\;\Psi_{l}(y,z|y^{\prime },z)/\eta ]dl \end{array}$$

where $\zeta (y,z|\omega )=-\nabla^{2}\psi (y,z|\omega )$ is the component of vorticity in the *x* direction, and the subscript *n* denotes differentiation normal to the boundary of *C*, out of the flow field while the subscript *l* denotes differentiation along *C*. Furthermore, *G*(*y*,*z*|*y*′,*z*′) is the Green’s function for the Laplace equation; *Ω*(*y*,*z*|*y*′,*z*′) is the Green’s function for the Helmholtz equation; *Ψ*(*y*,*z*|*y*′,*z*′) is the Green’s function for Equation (1). Detailed derivation of Equation (3) can be referred to Tuck [5] and Sader *et al.* [6]. Note that the two unknown quantities in Equation (3) are *ζ*(*y*,*z*|*ω*) and *P*(*y*,*z*|*ω*). When the cantilever is immersed in the fluid, the path of integration in Equation (3) describes a cross section of the system. It is appropriate to use the two-dimensional free-space Green’s functions as follows:

(4) $$G(y,z|y^{\prime },z^{\prime })=[\text{log}R]/2\pi$$

(5) $$\Omega (y,z|y^{\prime },z^{\prime })=-K_{0}(\sigma R)/2\pi$$

(6) $$\Psi (y,z|y^{\prime },z^{\prime })=-[\text{log}R+K_{0}(\sigma R)]/2\pi \sigma^{2}$$

in which $R=\sqrt{{\left(y-y^{\prime }\right)}^{2}+{\left(z-z^{\prime }\right)}^{2}}$ and *K*_0_ is the modified Bessel function of the third kind.

**Figure 1:**
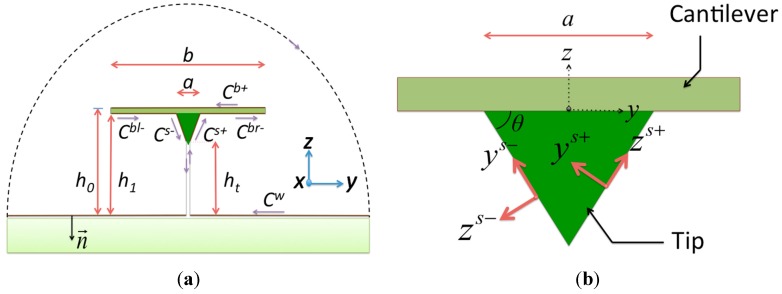
Cross sections of the system: (**a**) the integral path of the semi-analytical method and the global coordinate system and (**b**) the local coordinate system of the tip component.

In the present model, which includes the cantilever, tip, and specimen, we are interested in the hydrodynamic loading on the tip to the specimen. The integration path of a cross section of the system is shown in Figure 1a. For a cantilever with low thickness, it is apparent that the contour of integration is composed of three parts: the beam (beam top *C*^*b*+^ and beam bottom *C*^*b*−^), the surface of the tip (tip right *C*^*s*+^ and tip left *C*^*s*−^), and the planar specimen surface *C^w^*. Since the surfaces of the tip and specimen are assumed to be no-slip, the terms *ψ* and *ψ_n_* are zero. Equation (3) becomes:

(7) $$\begin{array}{ll} \psi & =\int_{c^{w}}\left[-\zeta^{w}\Psi_{n}+\frac{1}{\eta }P^{w}\Psi_{l}\right]\;dl+\int_{c^{b+}}\left[-\zeta^{b+}\Psi_{n}+\frac{1}{\eta }P^{b+}\Psi_{l}\right]dl+\int_{c^{b-}}\left[-\zeta^{b-}\Psi_{n}+\frac{1}{\eta }P^{b-}\Psi_{l}\right]dl\\ & +\int_{c^{s+}}\left[-\zeta^{s+}\Psi_{n}+\frac{1}{\eta }P^{s+}\Psi_{l}\right]\;dl+\int_{c^{s-}}\left[-\zeta^{s-}\Psi_{n}+\frac{1}{\eta }P^{s-}\Psi_{l}\right]\;dl \end{array}$$

in which (*ζ^w^*, *ζ^b+^*, *ζ*^*b*−^, *ζ^s^*^+^, and *ζ^s^*^−^) and (*P^w^*, *P^b^*^+^, *P^b^*^−^, *P^s^*^+^, and *P^s^*^−^) are the vorticity and the pressures, respectively, on the surface of the specimen, the beam top, the beam bottom, the tip right, and the tip left. The paths *C^w^*, *C*^*b*+^, and *C*^*b*−^ follow the global coordinate system (*y*,*z*) and the paths *C*^*s*+^ and *C*^*s*−^ should be transformed to local coordinate systems (*y*_*s*+_,*z*_*s*+_) and (*y*_*s*−_,*z*_*s*−_), as shown in Figure 1b. The path along the surface of the specimen is assumed to be infinite when compared with the tip scale, and so *C^w^* extends from *y* = −*∞* to *y* = *∞*. On *C^w^*, we have the differentiations *dl* = −*dy*, ∂/∂*l* = −∂/∂*y*, and ∂/∂*n* = −∂/∂*z*. In Figure 1b, the path *C*^*b*+^ extends from *y* = −*b*/2 to *y* = *b*/2, and the path *C*^*b*−^ separates into two parts: (1) *C^bl^*^−^ from *y* = −*b*/2 to *y* = −*a/2* and (2) *C^br^*^-^ from *y* = +*a/2* to *y* = *b*/2. We have the differentiations *dl* = *dy*, ∂/∂*l* = ∂/∂*y*, and ∂/∂*n* = ∂/∂*z* on *C*^*b*−^, and *dl* = −*dy*, ∂/∂*l* = −∂/∂*y*, and ∂/∂*n* = −∂/∂*z* on *C*^*b*+^. Furthermore, for the *C*^*s*+^ path, the transformation follows *y* = *y*_*s*+_cos*θ* − *z*_*s*+_sin*θ* and *z* = *y*_*s*+_cos*θ* + *z*_*s*+_sin*θ* + *h_t_*, where *h_t_* is the shortest distance from the top of the tip to the specimen. The integration along *y*_*s*+_ is from *y*_*s*+_ = 0 to *y*_*s*+_ = *d*, where *d* is the inclined length of the tip. We have the differentiations *dl* = *dy_s_*_+_, ∂/∂*l* = ∂/∂*y*_*s*+_, and ∂/∂*n* = ∂/∂*z*_*s*+_ on *C*^*s*+^. For the *C*^*s*−^ path, the transformation follows *y* = *y*_*s*−_cos*α* − *z*_*s*−_sin*α* and *z* = *y*_*s*−_cos*α* + *z*_*s*−_sin*α* + *h_t_*. The integration along *y*_*s*−_ is from *y*_*s*−_ = 0 to *y*_*s*−_ = *d*. We have the differentiations *dl* = −*dy_s_*_−_, ∂/∂*l* = −∂/∂*y*_*s*−_, and ∂/∂*n* = −∂/∂*z*_*s*−_ on *C^s+^*. With the above, Equation (7) can be rewritten as:

(8) ψ=∫−∞∞[ζwΨz−1ηPwΨy]dy+∫−b2b2[ζb+Ψz−1ηPb+Ψy]dy+∫−b2−a[−ζbl−Ψz+1ηPbl−Ψy]dy+∫ab2[−ζbr−Ψz+1ηPbr−Ψy]dy+∫0d[−ζs+Ψzs++1ηPs+Ψys+]dys++∫0d[ζs−Ψzs−−1ηPs−Ψys−]dys−

Differentiating Equation (8) with respect to to *z'* and *y'* yields the velocity components *v* and *w*. This allows us to evaluate each integral equation at the specimen surface (*z'* = 0), the beam surfaces (*z'* = *h*_0_ and *z'* = *h*_1_, where *h*_0_ and *h*_1_ are the locations of the beam top and beam bottom), and the tip surfaces (${z^{\prime }}_{s+}=h_{t}+{y^{\prime }}_{s+}\text{sin}\theta$ and ${z^{\prime }}_{s+}=h_{t}+{y^{\prime }}_{s+}\text{sin}\theta$). The 12 coupled integral equations for the velocity components *v* and *w* are listed in Appendix A. Although we only consider the bending mode, Equation (8) can be adapted to any AFM operating mode, such as the bending mode or the torsional mode.

The normal displacements on the surfaces of the beam and the tip are assumed to be a constant, *W*(*y*′|*ω*) = *W*_0_, and the displacement on the surface of the specimen is assumed to be zero. There are 12 boundary conditions. For the horizontal velocities on the surfaces, the following must be satisfied:

(9) $$\begin{array}{l} v(y^{\prime },0|\omega )=0\;\;\text{for}\;\;-\infty \leq y^{\prime }\leq \infty \\ v(y^{\prime },h_{0}|\omega )=0\;\;\text{for}\;\;-b/2\leq y^{\prime }\leq b/2\\ v(y^{\prime },h_{1}|\omega )=0\;\;\text{for}\;\;-b/2\leq y^{\prime }\leq -a/2\\ v(y^{\prime },h_{1}|\omega )=0\;\;\text{for}\;\;a/2\leq y^{\prime }\leq b/2\\ v(y_{s-}^{\prime },h_{t}+y_{s-}^{\prime }\text{sin}\theta |\omega )=0\;\;\text{for}\;\;0\leq y_{s-}^{\prime }\leq d\\ v(y_{s+}^{\prime },h_{t}+y_{s+}^{\prime }\text{sin}\theta |\omega )=0\;\;\text{for}\;\;0\leq y_{s+}^{\prime }\leq d \end{array}$$

For the vertical velocities on the surfaces, the following must be satisfied:

(10) $$\begin{array}{l} w(y^{\prime }0|\omega )=0\;\;\text{for}\;\;-\infty \leq y^{\prime }\leq \infty \\ w(y^{\prime },h_{0}|\omega )=W_{0}\;\;\text{for}\;\;-b/2\leq y^{\prime }\leq b/2\\ w(y_{L}^{\prime },h_{1}|\omega )=W_{0}\;\;\text{for}\;\;-b/2\leq y^{\prime }\leq -a/2\\ w(y_{R}^{\prime },h_{1}|\omega )=W_{0}\;\;\text{for}\;\;a/2\leq y^{\prime }\leq b/2\\ w(y_{s-}^{\prime },h_{t}+y_{s-}^{\prime }\text{sin}\theta |\omega )=W_{0}\;\;\text{for}\;\;0\leq y_{s-}^{\prime }\leq d\\ w(y_{s+}^{\prime },h_{t}+y_{s+}^{\prime }\text{sin}\theta |\omega )=W_{0}\;\;\text{for}\;\;0\leq y_{s+}^{\prime }\leq d \end{array}$$

From Equations (A1) to (A12) in the Appendix, Equations (9) and (10) can be simplified to a matrix notation:

(11) **A**·**X** = **F**

in which:

(12) $$A=\left[\begin{array}{ccc} A_{1,1} & \cdots & A_{1,12}\\ \vdots & \ddots & \vdots \\ A_{12,1} & \cdots & A_{12,12} \end{array}\right]$$

(13) $$X={\left\{\begin{array}{cccccccccccc} \zeta^{w} & P^{w} & \zeta^{b+} & P^{b+} & \zeta^{bl-} & P^{bl-} & \zeta^{br-} & P^{br-} & \zeta^{s+} & P^{s+} & \zeta^{s-} & P^{s-} \end{array}\right\}}^{T}$$

(14) $$F={\left\{\begin{array}{cccccccccccc} 0 & 0 & 0 & W_{0} & 0 & W_{0} & 0 & W_{0} & 0 & W_{0} & 0 & W_{0} \end{array}\right\}}^{T}$$

In addition, following Tuck’s dimensionless definition [5], the dimensionless length is *ξ* = 2*y*/*b*, the dimensionless pressure is $\bar{P}=b/(2\eta W_{0})P$, and the dimensionless vorticity is $\bar{\zeta }=(b/2W_{0})\zeta$. Furthermore, the Reynolds number of the flow is defined as *Re*(*ω*) = *ρωb*^2^/4*η*, which describes the relative importance of the linear inertial and viscous terms in the unsteady Stokes equation. In addition, for a beam undergoing normal oscillations in a viscous fluid, the general form of the hydrodynamic force (Fourier–transformed) per unit length in the *z* direction is given by:

(15) $$F_{z}(\omega )=-\pi i\rho \omega b^{2}W_{0}\Gamma^{n}(\omega )/4$$

in which the function Γ^*n*^—a complex-valued function whose real and imaginary parts are proportional to the inertial and viscous loading—is defined by:

(16) $$\Gamma^{n}(\omega )=\frac{i}{\pi \text{Re}(\omega )}\int_{-b/2}^{b/2}\Delta \bar{P}(y)dy$$

and $\Delta \bar{P}$ is the difference in pressure between the top and bottom of the beam.

## 3. Numerical Results

We select four different tip types to examine the geometric effects of the tips. These are the sharp model, the sine model, the cone model, and the bell model; their geometric properties are listed in Table 1. The sharp model and the cone model are typical models in commercial products, while the sine and bell models simulate the blunt tips after the tips are overused. The two key perspectives we consider are the vorticity and pressure induced by the effect of the tip shapes and by the tip–specimen distance.

**Table 1:** The different types of tip geometry.

Tip Types	Appearance
Cone shape (3 singularities) $z=\pm y+h_{t}$	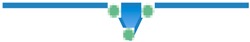
Bell shape (2 singularities) $\frac{y^{2}}{{\left(a/2\right)}^{2}}+\frac{{\left(z-h_{0}\right)}^{2}}{{\left(h_{0}-h_{t}\right)}^{2}}=1$	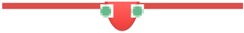
Sharp shape (1 singularity) ${\left(a/2\right)}^{2}={\left[y+{\left(a/2\right)}^{2}\right]}^{2}+{\left(z-h_{t}\right)}^{2}$	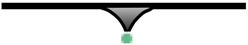
Sine shape $\begin{array}{l} y=t+1.5\pi ,\;z=\text{sin}\;t\\ -\pi \leq t\leq \pi \end{array}$	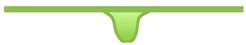

**Figure 2:**
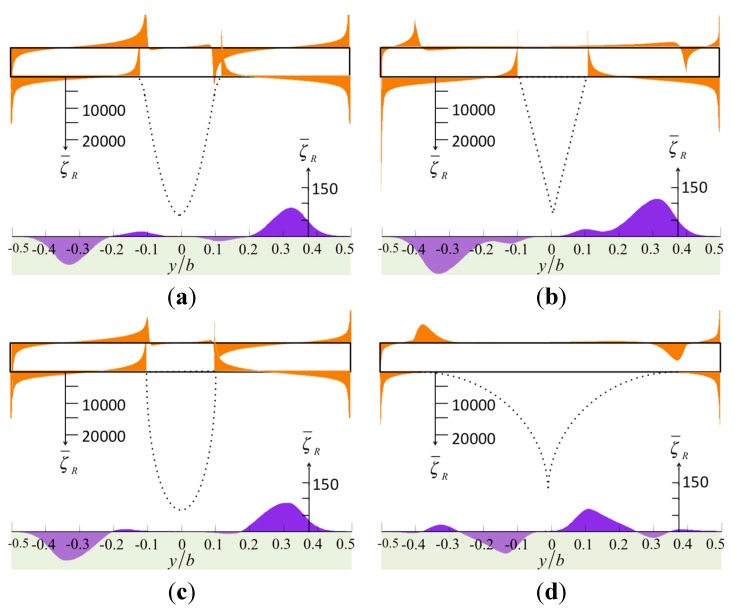
The normalized vorticity in real part over the surfaces of the cantilever and the specimen caused by various tip shapes: (**a**) the sharp model; (**b**) the sine-shaped model; (**c**) the cone-shaped model; and (**d**) the bell-shaped model.

**Figure 3:**
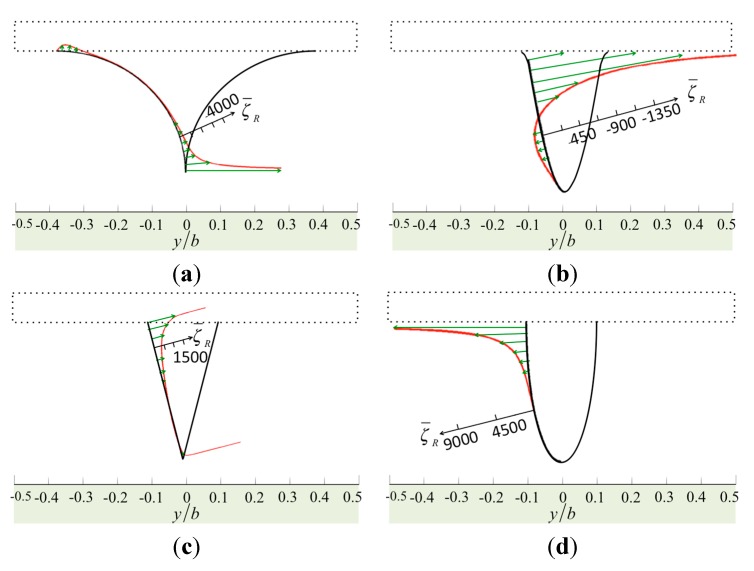
The normalized vorticity in real part over the surfaces of the tips: (**a**) the sharp model; (**b**) the sine-shaped model; (**c**) the cone-shaped model; and (**d**) the bell-shaped model.

When a tip is close to a specimen, the geometry of the tip dominates the degree of vorticity and pressure distribution over the surfaces. Figure 2 shows the normalized vorticity on the surface of the cantilever and the specimen, and Figure 3 shows the normalized vorticity on the tip. Here, the gap between the tip and the specimen is small, *h_t_*/*a* = 0.1. The tip is (*h*_1_ − *h_t_*)/*a* = 2.0 in height and *a/b* = 0.2 in width, and the Reynolds number is *Re* = 1 at low oscillation frequency or on a narrow cantilever. The fluid between the cantilever and the specimen may flow in a horizontal direction when the cantilever vibrates vertically. Thus, the streamline becomes intermingled, increasing the vorticity. In Figure 2, the results from the four tip models are similar, and large vorticity occurs near the corners. In Figure 3, we find a dramatic change when we compare the distribution from the sine model to that of the bell model. The vorticity of the sine model is much less than that of the bell model because the geometric curve of the sine tip has one inflection point and two concaves, and so the vorticity distribution along the tip has positive and negative parts. In addition, when we compare the bell model with the cone model, the angle between the tip base and the cantilever dominates the value of the vorticity. The cone model has a large angle, reducing the vorticity. Moreover, the sharp model has large vorticity at its tip end.

**Figure 4:**
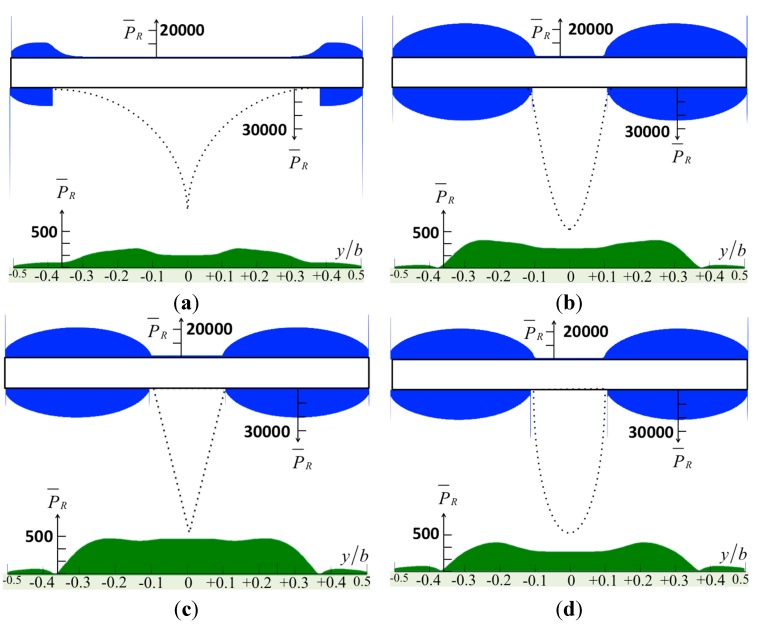
The normalized pressure in real part over the surfaces of the cantilever caused by various tip shapes: (**a**) the sharp model; (**b**) the sine-shaped model; (**c**) the cone-shaped model; and (**d**) the bell-shaped model.

**Figure 5:**
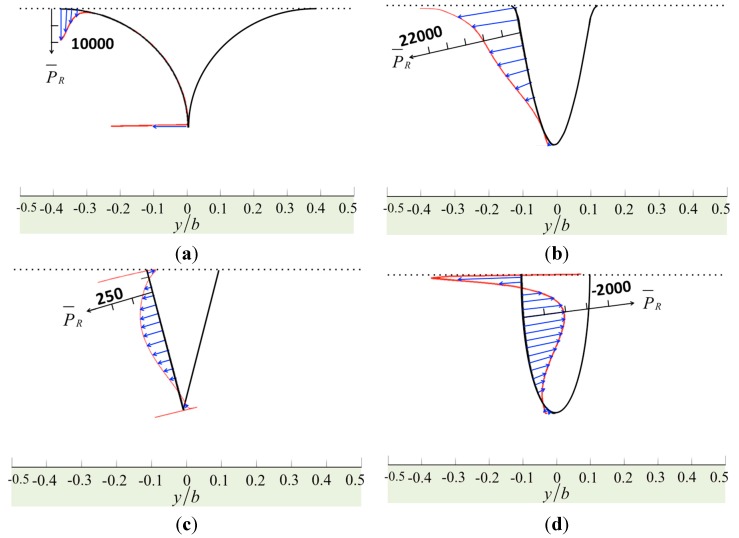
The normalized pressure in real part over the surfaces of the tips: (**a**) the sharp model, (**b**) the sine-shaped model; (**c**) the cone-shaped model; and (**d**) the bell-shaped model.

The tapping mode AFM with the stationary approach can be understood as thermodynamic cycles. Energy is transferred from a mechanical reservoir (cantilever oscillation) to a thermal reservoir (bulk specimen, tip, and surrounding liquid). Then, the energy dissipated per tapping cycle can be considered as the net energy density integrated over the interaction area. The normalized pressure distributions on the surfaces of the cantilever, the tip, and the specimen are shown in Figure 4 and Figure 5. The results show that the sharp tip applies less pressure to the specimen. On average, the major region where the energy transferred from the tip in to the bulk specimen is three times the tip base width (~3*a*), excluding the sharp model. The maximum pressure on the specimen, as shown in Figure 4, is $\bar{P}=\sim 500$, which may deform an erythrocyte, for example, thus reducing the measurement resolution. Accordingly, $P=(2\eta W_{0})\bar{P}/b$, and we define $\eta =1\times 10^{-3}$, so that we have *P* = *W*_0_/*b*. From these figures, the pressure is distributed evenly over 60% of the dimensionless width of the cantilever. Janovjak *et al.* [20] presented an approach for estimating the hydrodynamic effects, thereby allowing an evaluation of AFM force measurements recorded over an extended range of pulling speeds. Here, we compare our results with their study. The width of the cantilever *b* = 18 μm, and so 60% of the width is 10.8 μm. Thus, the main pressure area is *A* = 116.64 μm^2^. Then, the force is *F* = *PA* = *W*_0_*A*/*b* = 6.48*W*_0_ pN, and the unit of *W*_0_ is μm/s. This result agrees with the drag forces measured by AFM cantilevers, as shown in Figure 2a of study [20].

According to $\bar{P}=\sim 500$ in Figure 4, we assume *W*_0_ =1 and *b* = 2, so that *P* = ~0.5 Pa. If we give a typical cantilever *b* = 50 μm, and then the force is *F* = 0.45 nN. To determine how much the tip affects a specimen without tapping, we simply apply the equation of Hertz model $dz\;=\;{\left[3F/(4\sqrt{R_{c}}E*)\right]}^{2/3}$ to calculate the hydrodynamic indentation depth. Here, we assume the radius of curvature of the pressure is *R_c_* = 15 μm. Additionally, the relative Young’s modulus E* is defined as 1/E* = (1− *γ*_1_^2^)/*E*_1_ + (1 − *γ*_2_^2^)/*E*_2_ ≅ (1 − *γ*_1_^2^)/*E*_1_ for *E*_1_ << *E*_2_. Here *E*_1_, *E*_2_, *γ*_1_, and *γ*_2_ are the Young’s moduli and Poisson ratios for the specimen and the tip, respectively. Note that *E*_2_ for the tip is ~150 GPa (a Si_3_N_4_ tip) greater than that of the bio-specimen *E*_1_ = 4.4 kPa (using the example of an erythrocyte) and *γ*_1_ is 0.49. As a result, the E* = 5.79 kPa, and the hydrodynamic indentation depth of the erythrocyte is calculated to be 61 nm. Further, Möller *et al.* [16] measured the averaged heights of the topography of the extracellular purple membrane surfaces measured from the tapping mode and found that there were ~25% smaller than those measured by the contact mode. This example provides an opportunity to discuss the indentation depth. In the case study, the stiffness of the membrane is *k* = 1 N/m [17]; the height difference of the maximum protrusion over the lipid bilayer is *h* = 0.5 nm [16]; the tip radius is assumed to be *r* = 2 nm; and the Reynolds number is assumed to be *Re* = 1. Thus, *E*_1_ is approximately 39.8 MPa (*E*_1_ = k*h/π*r*^2^), and the indentation depth of the membrane is 0.134 nm. This rough estimation could be applied to explain why the heights of membranes measured by the tapping mode are ~0.1 nm smaller than those measured by the contact mode in the study of Möller *et al.*

Medalsy and Müller [17] showed that the stiffness of the membrane depends on the tip velocity. Higher tip velocity increases the membrane stiffness. This phenomenon has been observed on extracellular/cytoplasmic purple membrane surfaces. The hydrodynamic effect changes the apparent AFM stiffness, and the hydrodynamic force is linearly proportional to the tip velocity. Therefore, the hydrodynamic force could be obtained by multiplying the pressure *P* by the contact area *A_c_*, *i.e.*, $F_{D}=(2\eta A_{c}\bar{P}/b)W_{0}$. In the determination of the membrane biomechanical properties, the overall mechanical stiffness, *k*_total_, consists of a structural attribution to the protein membrane *k*_PM_ and an electrostatic attribution to the electrostatic double layer repulsion *k*_EL_.

(17) $$k_{total}=k_{PM}k_{EL}/(k_{PM}+k_{EL})$$

However, the apparent force should include the hydrodynamic force from the AFM cantilever force *F*_AFM_:

(18) $$F_{AFM}=F_{D}+k_{total}\Delta =2\eta \bar{P}W_{0}A_{c}/b+k_{PM}k_{EL}/(k_{PM}+k_{EL})\Delta$$

where Δ is the indentation. To study the effective cell stiffness, a uniform radial pre-stress is prescribed throughout the cytoplasm, normally 0.5 kPa. The simultaneous equilibration indentation process of the AFM tip generally considers both pre-stress and AFM indentation force [19]. Here, the authors claim that the hydrodynamic pressure needs to be considered in the simultaneous equilibration.

**Figure 6:**
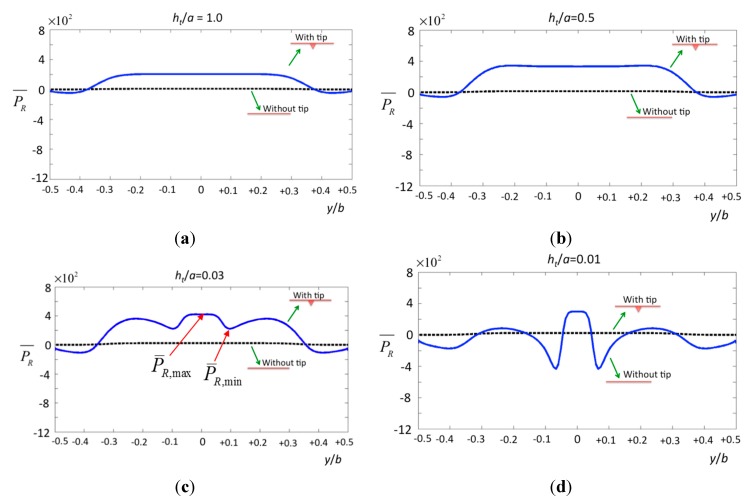
The normalized pressure in real part over the surface of the specimen by a cone tip with various tip–specimen distances (**a**) *h_t_*/*a* = 1.0; (**b**) *h_t_*/*a* = 0.5; (**c**) *h_t_*/*a* = 0.03; and (**d**) *h_t_*/*a* = 0.01.

In addition, AFM is a promising approach to measuring mechanisms involved in deterioration and refurbishment of the endothelial glycocalyx. Recently, AFM indentation was used detect the thickness and stiffness of a glycocalyx. The tip velocity is generally ~1 μm/s, and the hydrodynamic force is calculated as 6.8 pN (using our previous result F = 6.48*W*_0_ pN). This force is the pre-stress force applied to the glycocalyx before the tip contacts it, and the indentation depth is roughly obtained as 17.9 nm (the endothelial glycocalyx stiffness is 0.38 pN/nm [21]). The indentation depth is around 4% of the thickness of glycocalyx. The vorticity describes the maximum spinning motion of the glycocalyx in the area 0.1 < |*y*/*b*| < 0.4. When the tip is downward, the shear force induced by the vorticity bends the glycocalyx in the direction away from the tip, and the force is too small to damage the glycocalyx.

For the pressure according to various tip–specimen distances, the cone-shaped tip causes a rapid change of pressure on the surface of the specimen. To indicate the contribution from the tip, the solid line in Figure 6 shows that the specimen bears considerable pressure inside the region −0.5 ≤ *y*/*b* ≤ 0.5, and the pressure drops markedly at *y*/*b* = ±0.1, which is the region containing the tip. On the other hand, the dashed line shows the pressure resulting from the cantilever without the tip. Obviously, the dramatic changes in the non-uniform pressure may deform the surface of a specimen. Regardless of distance, the pressure of the cantilever with the tip (solid lines) is around 20 times larger than that of the cantilever without considering the tip as shown in figure (dashed lines). This is the key contribution of our paper, particularly compared with previous studies [6,7,8,9,10,11], which did not consider the tip effect.

**Figure 7:**
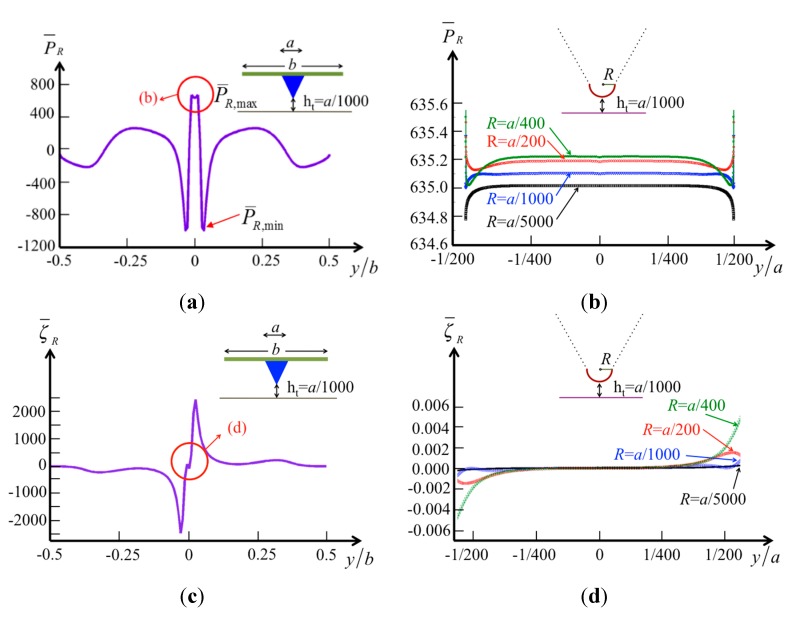
The normalized pressure and vorticity in real part over the surface of the specimen by a cone tip at a constant tip height *h_t_*/*a* = 0.001 (**a**) pressure in global region; (**b**) pressure in local region with various tip radii; (**c**) vorticity in global region; and (**d**) vorticity in local region with various tip radii.

To analyze the effect of the tip radius on the pressure, we calculate the pressure and vorticity distributions on the surface of the specimen. Here, the tip–specimen distance is fixed as a reference, *ht* = *a*/1000 (where *a* is the tip width, and we assume *a* = 10 μm and *b* = 50 μm), and the radii of the tip are *a*/200, *a*/400, *a*/1000, and *a*/5000 (*i.e*., 50 nm, 25 nm, 10 nm, and 2 nm, as typically used). The pressure along the beam width *b* on the surface is demonstrated in Figure 7a. When Figure 7a is compared with Figure 6c,d, it is obvious that the central influence area shrinks (from |*y*/*b*| < 0.1 to |*y*/*b*| < 1/200) with decreasing tip–specimen distance (from *ht*/*a* = 0.03 to *ht*/*a* = 0.001). However, the pressure difference $\Delta {\bar{P}}_{R}={\bar{P}}_{R,\text{max}}-{\bar{P}}_{R,\text{min}}$ in the central influence area increases (from $\Delta {\bar{P}}_{R}$ = 200–1700) with the same decreasing tip–specimen distance. There exists a region where the pressure changes rapidly from positive to negative (|*y*/*b*| < 1/16). The vorticity distribution also shows the rapid change in this region, where the fluid in both sides of the tip may flow in opposite directions when the tip vibrates vertically. When we consider the effects of various tip radii, the results shown in Figure 7b indicate small changes of the hydrodynamic pressure in this narrow region (−1/200 < *y/a* < 1/200). The vorticity distribution shown in Figure 7d demonstrates zero vorticity in the region (−1/400 < *y/a* < 1/400) for various tip radii.

**Figure 8:**
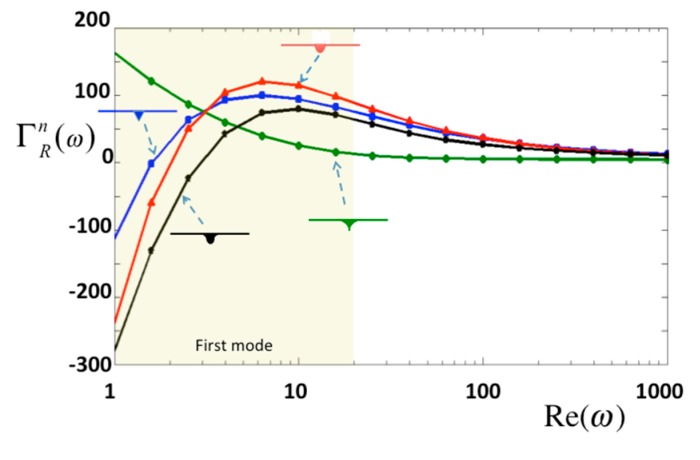
Normalized hydrodynamic force in real part per unit length for normal oscillation.

Moreover, oscillating modes are useful for investigating the viscous properties of materials. In this study, the oscillating frequencies are incorporated into the Reynolds number [*Re*(*ω*) = *ρωb*^2^/4*η*]. Note that *η* is the viscosity of the surrounding material. In this study, the normalized hydrodynamic forces *Γ^n^* against the Reynolds numbers are demonstrated in Figure 8, in which *h_t_*/*a* = 0.1. Four different types of tips are compared, and the results shows that three tips produce a rapid change in 1 < *Re* < 6, excluding the sharp tip which also has a small pressure on the tip as shown in Figure 5a. Note that the first mode of a typical bio-cantilever is in the region, 1 < *Re* < 20, where *Γ^n^* changes rapidly.

## 4. Conclusions

In a liquid environment, an AFM probe operating in the tapping mode affects the system not only by shifting the resonance frequency of the cantilever but also by the tip itself inducing hydrodynamic pressure on the specimen. We used a semi-analytical method to analyze the pressure and vorticity distribution caused by various tip geometries. The results show that the geometric curvature of the tip leads to dramatic changes in vorticity and pressure on the tip surface. When the tip moves near the specimen, the pressure on the surface of the specimen changes rapidly. At a height of *h_t_*/*a* = 0.1, the hydrodynamic pressure induced by the cone-shaped tip and applied to the specimen surface is ~0.5 Pa, which may pre-compress an erythrocyte cell by 61 nm and an extracellular membrane by 0.134 nm. The tip velocity also affects the stiffness of a membrane, and the apparent force of AFM may reduce the hydrodynamic force. When the tip nears the specimen at *h_t_*/*a*<0.03, the pressure on the surface of the specimen changes rapidly; when *h_t_*/*a* = 0.001, the hydrodynamic pressure can be considered a concentrated pressure applied to the specimen in region −1/32 < *y*/*b* < 1/32. Regardless of the tip–specimen distance, the pressure of the cantilever with the tip is around 20 times larger than that of the cantilever without considering the tip. Furthermore, the radius of the tip does not affect the hydrodynamic pressure in the case where the specimen is assumed to be planar.

## Appendix A

(a) For the observation points located at *C^w^*, the velocities in the *y* and *z* directions are respectively:

(A1) v(y′,0|ω)=∫∞−∞[ζwΨzz′(y,0|y′,0)−1ηpwΨyz′(y,0|y′,0)]dy+∫−b2b2[ζb+Ψzz′(y,h0|y′,0)−1ηpb+Ψyz′(y,h0|y′,0)]dy+∫−b2−a2[−ζbl−Ψzz′(y,h1|y′,0)+1ηpbl−Ψyz′(y,h1|y′,0)]dy+∫a2b2[−ζbr−Ψzz′(y,h1|y′,0)+1ηpbr−Ψyz′(y,h1|y′,0)]dy+∫0d[−ζs+Ψzs+zs+′(ys+,0|ys+′,0)+1ηps+Ψys+zs+′(ys+,0|ys+′,0)]dzs+′×cosθ+∫0d[ζs+Ψzs+ys+′(ys+,0|ys+′,0)−1ηps+Ψys+ys+′(ys+,0|ys+′,0)]dys+′×(−sinθ)+∫0d[ζs−Ψzs−zs−′(ys−,0|ys−′,0)−1ηps−Ψys−zs−′(ys−,0|ys−′,0)]dzs−′×cosα+∫0d[−ζs−Ψzs−ys−′(ys−,0|ys−′,0)+1ηps−Ψys−ys−′(ys−,0|ys−′,0)]dys−′×(−sinα)

and:

(A2) w(y′,0|ω)=∫∞−∞[−ζwΨzy′(y,0|y′,0)+1ηPwΨyy′(y,0|y′,0)]dy+∫−b2b2[−ζb+Ψzy′(y,h0|y′,0)+1ηpb+Ψyy′(y,h0|y′,0)]dy+∫−b2−a2[ζbl−Ψzy′(y,h1|y′,0)−1ηpbl−Ψyy′(y,h1|y′,0)]dy+∫a2b2[ζbr−Ψzy′(y,h1|y′,0)−1ηpbr−Ψyy′(y,h1|y′,0)]dy+∫0d[−ζs+Ψzs+zs+′(ys+,0|ys+′,0)+1ηps+Ψys+zs+′(ys+,0|ys+′,0)]dzs+′×sinθ+∫0d[ζs+Ψzs+ys+′(ys+,0|y′,0)−1ηps+Ψys+ys+′(ys+,0|y′,0)]dys+′×cosθ+∫0d[ζs−Ψzs−zs−′(ys−,0|ys−′,0)−1ηps−Ψys−zs−′(ys−,0|ys−′,0)]dzs−′×sinα+∫0d[−ζs−Ψzs−ys−′(ys−,0|ys−′,0)+1ηps−Ψys−ys−′(ys−,0|ys−′,0)]dzs−′×cosα

(b) For the observation points are located at *C*^*b*+^, the velocities in the *y* and *z* directions are respectively:

(A3) v(y′,h1|ω)=∫∞−∞[ζwΨzz′(y,0|y′,h0)−1ηpwΨyz′(y,0|y′,h0)]dy+∫−b2b2[ζb+Ψzz′(y,h0|y′,h0)−1ηpb+Ψyz′(y,h0|y′,h0)]dy+∫−b2−a2[−ζbl−Ψzz′(y,h1|y′,h0)+1ηpbl−Ψyz′(y,h1|y′,h0)]dy+∫a2b2[−ζbr−Ψzz′(y,h1|y′,h0)+1ηpbr−Ψyz′(y,h1|y′,h0)]dy+∫0d[−ζs+Ψzs+zs+′(ys+,0|ys+′,h0)+1ηps+Ψys+zs+′(ys+,0|ys+′,h0)]dzs+′×cosθ+∫0d[ζs+Ψzs+ys+′(ys+,0|ys+′,h0)−1ηps+Ψys+ys+′(ys+,0|ys+′,h0)]dys+′×(−sinθ)+∫0d[ζs−Ψzs−zs−′(ys−,0|ys−′,h0)−1ηps−Ψys−zs−′(ys−,0|ys−′,h0)]dzs−′×cosα+∫0d[−ζs−Ψzs−ys−′(ys−,0|ys−′,h0)+1ηps−Ψys−ys−′(ys−,0|ys−′,h0)]dys−′×(−sinα)

and:

(A4) w(y′,h1|ω)=∫∞−∞[−ζwΨzy′(y,0|y′,h0)+1ηpwΨyy′(y,0|y′,h0)]dy+∫−b2b2[−ζb+Ψzy′(y,h0|y′,h0)+1ηpb+Ψyy′(y,h0|y′,h0)]dy+∫−b2−a2[ζbl−Ψzy′(y,h1|y′,h0)−1ηpbl−Ψyy′(y,h1|y′,h0)]dy+∫a2b2[ζbr−Ψzy′(y,h1|y′,h0)−1ηpbr−Ψyy′(y,h1|y′,h0)]dy+∫0d[−ζs+Ψzs+zs+′(ys+,0|ys+′,h0)+1ηps+Ψys+zs+′(ys+,0|ys+′,h0)]dzs+′×sinθ+∫0d[ζs+Ψzs+ys+′(ys+,0|ys+′,h0)−1ηps+Ψys+ys+′(ys+,0|ys+′,h0)]dys+′×cosθ+∫0d[ζs−Ψzs−zs−′(ys−,0|ys−′,h0)−1ηps−Ψys−zs−′(ys−,0|ys−′,h0)]dzs−′×sinα+∫0d[−ζs−Ψzs−ys−′(ys−,0|ys−′,h0)+1ηps−Ψys−ys−′(ys−,0|ys−′,h0)]dys−′×cosα

(c) For the observation points located at *C^bl−^*, the velocities in the *y* and *z* directions are respectively:

(A5) v(y′,h1|ω)=∫∞−∞[ζwΨzz′(y,0|y′,h1)−1ηpwΨyz′(y,0|y′,h1)]dy+∫−b2b2[ζb+Ψzz′(y,h0|y′,h1)−1ηpb+Ψyz′(y,h0|y′,h1)]dy+∫−b2−a2[−ζbl−Ψzz′(y,h1|y′,h1)+1ηpbl−Ψyz′(y,h1|y′,h1)]dy+∫a2b2[−ζbr−Ψzz′(y,h1|y′,h1)+1ηpbr−Ψyz′(y,h1|y′,h1)]dy+∫0d[−ζs+Ψzs+zs+′(ys+,0|ys+′,h1)+1ηps+Ψys+zs+′(ys+,0|ys+′,h1)]dzs+′×cosθ+∫0d[ζs+Ψzs+ys+′(ys+,0|ys+′,h1)−1ηps+Ψys+ys+′(ys+,0|ys+′,h1)]dys+′×(−sinα)+∫0d[ζs−Ψzs−zs−′(ys−,0|ys−′,h1)−1ηps−Ψys−zs−′(ys−,0|ys−′,h1)]dzs−′×cosα+∫0d[−ζs−Ψzs−ys−′(ys−,0|ys−′,h1)+1ηps−Ψys−ys−′(ys−,0|ys−′,h1)]dys−′×(−sinθ)

and:

(A6) w(y′,h1|ω)=∫∞−∞[−ζwΨzy′(y,0|y′,h1)+1ηpwΨyy′(y,0|y′,h1)]dy+∫−b2b2[−ζb+Ψzy′(y,h0|y′,h1)+1ηpb+Ψyy′(y,h0|y′,h1)]dy+∫−b2−a2[ζbl−Ψzy′(y,h1|y′,h1)−1ηpbl−Ψyy′(y,h1|y′,h1)]dy+∫a2b2[ζbr−Ψzy′(y,h1|y′,h1)−1ηpbr−Ψyz′(y,h1|y′,h1)]dy+∫0d[−ζs+Ψzs+zs+′(ys+,0|ys+′,h1)+1ηps+Ψys+zs+′(ys+,0|ys+′,h1)]dzs+′×sinθ+∫0d[ζs+Ψzs+ys+′(ys+,0|ys+′,h1)−1ηps+Ψys+ys+′(ys+,0|ys+′,h1)]dys+′×cosθ+∫0d[ζs−Ψzs−zs−′(ys−,0|ys−′,h1)−1ηps−Ψys−zs−′(ys−,0|ys−′,h1)]dzs−′×sinα+∫0d[−ζs−Ψzs−ys−′(ys−,0|ys−′,h1)+1ηps−Ψys−ys−′(ys−,0|ys−′,h1)]dys−′×cosα

(d) For the observation points located at *C^br−^*, the velocities in the *y* and *z* directions are respectively:

(A7) v(y′,h1|ω)=∫∞−∞[ζwΨzz′(y,0|y′,h1)−1ηpwΨyz′(y,0|y′,h1)]dy+∫−b2b2[ζb+Ψzz′(y,h0|y′,h1)−1ηpb+Ψyz′(y,h0|y′,h1)]dy+∫−b2−a2[−ζbl−Ψzz′(y,h1|y′,h1)+1ηpbl−Ψyz′(y,h1|y′,h1)]dy+∫a2b2[−ζbr−Ψzz′(y,h1|y′,h1)+1ηpbr−Ψyz′(y,h1|y′,h1)]dy+∫0d[−ζs+Ψzs+zs+′(ys+,0|ys+′,h1)+1ηps+Ψys+zs+′(ys+,0|ys+′,h1)]dzs+′×cosθ+∫0d[ζs+Ψzs+ys+′(ys+,0|ys+′,h1)−1ηps+Ψys+ys+′(ys+,0|ys+′,h1)]dys+′×(−sinθ)+∫0d[ζs−Ψzs−zs−′(ys−,0|ys−′,h1)−1ηps−Ψys−zs−′(ys+,0|ys−′,h1)]dzs−′×cosα+∫0d[−ζs−Ψzs−ys−′(ys−,0|ys−′,h1)+1ηps−Ψys−ys−′(ys+,0|ys−′,h1)]dys−′×(−sinα)

and:

(A8) w(y′,h1|ω)=∫∞−∞[−ζwΨzy′(y,0|y′,h1)+1ηpwΨyy′(y,0|y′,h1)]dy+∫−b2b2[−ζb+Ψzy′(y,h0|y′,h1)+1ηpb+Ψyy′(y,h0|y′,h1)]dy+∫−b2−a2[ζbl−Ψzy′(y,h1|y′,h1)−1ηpbl−Ψyy′(y,h1|y′,h1)]dy+∫a2b2[ζbr−Ψzy′(y,h1|y′,h1)−1ηpbr−Ψyy′(y,h1|y′,h1)]dy+∫0d[−ζs+Ψzs+zs+′(ys+,0|ys+′,h1)+1ηps+Ψys+zs+′(ys+,0|ys+′,h1)]dzs+′×sinθ+∫0d[ζs+Ψzs+ys+′(ys+,0|ys+′,h1)−1ηps+Ψys+ys+′(ys+,0|ys+′,h1)]dys+′×cosθ+∫0d[ζs−Ψzs−zs−′(ys−,0|ys−′,h1)−1ηps−Ψys−zs−′(ys−,0|ys−′,h1)]dzs−′×sinα+∫0d[−ζs−Ψzs−ys−′(ys−,0|ys−′,h1)+1ηps−Ψys−ys−′(ys−,0|ys−′,h1)]dys−′×cosα

(e) For the observation points located at *C^s+^*, the velocities in the *y* and *z* directions are respectively:

(A9) v(ys+′,ht+ys+′×sinθ|ω)=∫∞−∞[ζwΨzz′(y,0|ys+′,ht+ys+′×sinθ)−1ηpwΨyz′(y,0|ys+′,ht+ys+′×sinθ)]dy+∫−b2b2[ζb+Ψzz′(y,h0|ys+′,ht+ys+′×sinθ)−1ηpb+Ψyz′(y,h0|ys+′,ht+ys+′×sinθ)]dy+∫−b2−a2[−ζbl−Ψzz′(y,h1|ys+′,ht+ys+′×sinθ)+1ηpbl−Ψyz′(y,h1|ys+′,ht+ys+′×sinθ)]dy+∫a2b2[−ζbr−Ψzz′(y,h1|ys+′,ht+ys+′×sinθ)+1ηpbr−Ψyz′(y,h1|ys+′,ht+ys+′×sinθ)]dy+∫0d[−ζs+Ψzs+zs+′(ys+,0|0,0)+1ηps+Ψys+zs+′(ys+,0|0,0)]dzs+′×cosθ+∫0d[ζs+Ψzs+ys+′(ys+,0|0,0)−1ηps+Ψys+ys+′(ys+,0|0,0)]dys+′×(−sinθ)+∫0d[ζs−Ψzs−zs−′(ys−,0|0,0)−1ηps−Ψys−zs−′(ys−,0|0,0)]dzs−′×cosα+∫0d[−ζs−Ψzs−ys−′(ys−,0|0,0)+1ηps−Ψys−ys−′(ys−,0|0,0)]dys−′×(−sinα)

and:

(A10) w(ys+′,ht+ys+′×sinθ|ω)=∫∞−∞[−ζwΨzz′(y,0|ys+′,ht+ys+′×sinθ)+1ηpwΨyz′(y,0|ys+′,ht+ys+′×sinθ)]dy+∫−b2b2[−ζb+Ψzy′(y,h0|ys+′,ht+ys+′×sinθ)+1ηpb+Ψyy′(y,h0|ys+′,ht+ys+′×sinθ)]dy+∫−b2−a2[ζbl−Ψzy′(y,h1|ys+′,ht+ys+′×sinθ)−1ηpbl−Ψyy′(y,h1|ys+′,ht+ys+′×sinθ)]dy+∫a2b2[ζbr−Ψzy′(y,h1|ys+′,ht+ys+′×sinθ)−1ηpbr−Ψyy′(y,h1|ys+′,ht+ys+′×sinθ)]dy+∫0d[−ζs+Ψzs+zs+′(ys+,0|0,0)+1ηps+Ψys+zs+′(ys+,0|0,0)]dzs+′×sinθ+∫0d[ζs+Ψzs+ys+′(ys+,0|0,0)−1ηps+Ψys+ys+′(ys+,0|0,0)]dys+′×cosθ+∫0d[ζs−Ψzs−zs−′(ys−,0|0,0)−1ηps−Ψys−zs−′(ys−,0|0,0)]dzs−′×sinα+∫0d[−ζs−Ψzs−ys−′(ys−,0|0,0)+1ηps−Ψys−ys−′(ys−,0|0,0)]dys−′×cosα

(f) For the observation points located at *C^s−^*, the velocities in the *y* and *z* directions are respectively:

(A11) v(ht+ys−′×sin(α−90)|ω)=∫∞−∞[ζwΨzz′(y,0|ys−′,ht+ys−′×sin(α−90))−1ηpwΨyz′(y,0|ys−′,ht+ys−′×sin(α−90))]dy+∫−b2b2[ζb+Ψzz′(y,h0|ys−′,ht+ys−′×sin(α−90))−1ηpb+Ψyz′(y,h0|ys−′,ht+ys−′×sin(α−90))]dy+∫−b2−a2[−ζbl−Ψzz′(y,h1|ys−′,ht+ys−′×sin(α−90))+1ηpbl−Ψyz′(y,h1|ys−′,ht+ys−′×sin(α−90))]dy+∫a2b2[−ζbr−Ψzz′(y,h1|ys−′,ht+ys−′×sin(α−90))+1ηpbr−Ψyz′(y,h1|ys−′,ht+ys−′×sin(α−90))]dy+∫0d[−ζs+Ψzs+zs+′(ys+,0|0,0)+1ηps+Ψys+zs+′(ys+,0|0,0)]dzs+′×cosθ+∫0d[ζs+Ψzs+ys+′(ys+,0|0,0)−1ηps+Ψys+ys+′(ys+,0|0,0)]dys+′×(−sinθ)+∫0d[ζs−Ψzs−zs−′(ys−,0|0,0)−1ηps−Ψys−zs−′(ys−,0|0,0)]dzs−′×cosα+∫0d[−ζs−Ψzs−ys−′(ys−,0|0,0)+1ηps−Ψys−ys−′(ys−,0|0,0)]dys−′×(−sinα)

and:

(A12) w(ht+ys−′×sin(α−90)|ω)=∫∞−∞[−ζwΨzy′(y,0|ys−′,ht+ys−′×sin(α−90))+1ηpwΨyy′(y,0|ys−′,ht+ys−′×sin(α−90))]dy+∫−b2b2[−ζb+Ψzy′(y,h0|ys−′,ht+ys−′×sin(α−90))+1ηpb+Ψyy′(y,h0|ys−′,ht+ys−′×sin(α−90))]dy+∫−b2−a2[ζbl−Ψzy′(y,h1|ys−′,ht+ys−′×sin(α−90))−1ηpbl−Ψyy′(y,h1|ys−′,ht+ys−′×sin(α−90))]dy+∫a2b2[ζbr−Ψzy′(y,h1|ys−′,ht+ys−′×sin(α−90))−1ηpbr−Ψyy′(y,h1|ys−′,ht+ys−′×sin(α−90))]dy+∫0d[−ζs+Ψzs+zs+′(ys+,0|0,0)+1ηps+Ψys+zs+′(ys+,0|0,0)]dzs+′×sinθ+∫0d[ζs+Ψzs+ys+′(ys+,0|0,0)−1ηps+Ψys+ys+′(ys+,0|0,0)]dys+′×cosθ+∫0d[ζs−Ψzs−zs−′(ys−,0|0,0)−1ηps−Ψys−zs−′(ys−,0|0,0)]dzs−′×sinα+∫0d[−ζs−Ψzs−ys−′(ys−,0|0,0)+1ηps−Ψys−ys−′(ys−,0|0,0)]dys−′×cosα
